# Nanomaterials as Promising Alternative in the Infection Treatment

**DOI:** 10.3390/ijms20153806

**Published:** 2019-08-04

**Authors:** María Vallet-Regí, Blanca González, Isabel Izquierdo-Barba

**Affiliations:** 1 Departamento de Química en Ciencias Farmacéuticas, Unidad de Química Inorgánica y Bioinorgánica, Universidad Complutense de Madrid, Instituto de Investigación Sanitaria Hospital 12 de Octubre i+12, Plaza Ramón y Cajal s/n, Madrid 28040, Spain; 2 CIBER de Bioingeniería, Biomateriales y Nanomedicina, CIBER-BBN, Madrid 28040, Spain

**Keywords:** preventing infection, zwitterionic surfaces, nanopattering surfaces, nanoparticles, mesoporous silica nanoparticles, antibacterial activity, targeting

## Abstract

Both the prevalence of antibiotic resistance and the increased biofilm-associated infections are boosting the demand for new advanced and more effective treatment for such infections. In this sense, nanotechnology offers a ground-breaking platform for addressing this challenge. This review shows the current progress in the field of antimicrobial inorganic-based nanomaterials and their activity against bacteria and bacterial biofilm. Herein, nanomaterials preventing the bacteria adhesion and nanomaterials treating the infection once formed are presented through a classification based on their functionality. To fight infection, nanoparticles with inherent antibacterial activity and nanoparticles acting as nanovehicles are described, emphasizing the design of the carrier nanosystems with properties targeting the bacteria and the biofilm.

## 1. Introduction

Currently, antimicrobial resistance (AMR) is pervasive across 22 countries with an estimated 500,000 people infected worldwide [1,2]. The macroeconomist, Peter O’Neill, has alerted all governments worldwide that deaths due to AMR will outpace cancer by 2050, estimating 10 million deaths by such date [3]. A consequence of uncontrolled bacterial growth is increased prevalence of biofilms, which are microorganisms’ communities typically composed of multiple species coated in a self-produced protective extracellular matrix [4]. Biofilms protect the integral bacteria, allowing them to survive in hostile surroundings, as their physiology and behavior are considerably different from their free-living counterparts (i.e., planktonic). In this way, biofilms confer resistance to antimicrobial agents and to the immune system, causing persistent and chronic infections. Undoubtedly, both AMR and biofilm formation constitute serious clinical problems and currently it is estimated that 60%–80% of chronic persistent infections treated in hospitals today are produced by bacterial biofilms [5].

Nanotechnology offers potential opportunities in many fields, including infectious processes [6,7]. The term “nano”, according to the Food and Drugs Administration (FDA) and International Union of Pure and Applied Chemistry (IUPAC), refers to any product with properties or phenomena attributable to its dimensions, when such dimensions are in the nanoscale range of 1–100 nm [8,9]. These nanomaterials have unique properties compared to their bulk chemical counterparts, such as large surface area to volume ratio and versatility, which could enhance their influence on a given microorganism and other diseases [10,11,12]. The advantage of these nanomaterial formulations over conventional systems is that they can increase treatment efficacy and decrease side effects through their precise targeting mode of action. In this sense, nanomedicine comprises the use of nanoparticles as therapeutic agents, drug delivery, and diagnosis systems, or the use of nanomaterials for medical devices [10]. In the last decade, many billions of dollars have been invested in the global market of nanomaterials for medicine, with particular interest in the area of drug delivery systems. Moreover, as a consequence there are some concerns over the potential toxicology of nanomaterials in the human environment being diverse and recent literature explores their possible adverse health effects [13].

**Figure 1:**
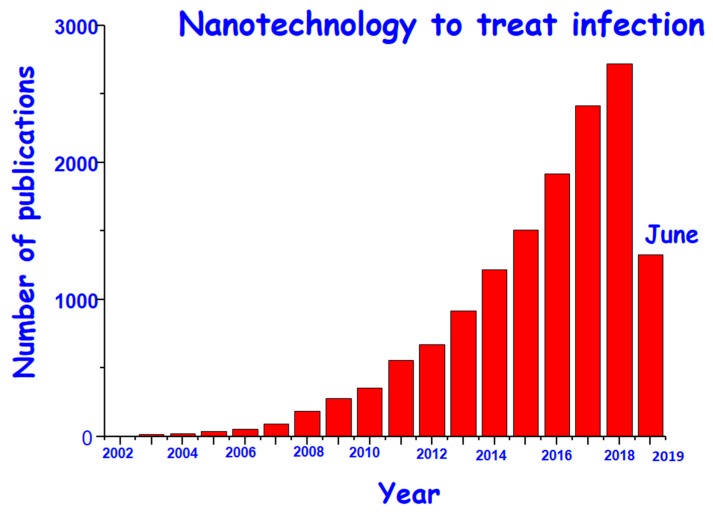
Histogram showing the research of nanomaterials for infection treatment from 2002 until today (June 2019). Database: Web of Science (WOS); and keywords: “Nanomaterials” and “infection”.

From the moment that nanomaterials were first described as drug delivery systems [14] much research effort has been made, especially focused on cancer treatment [15,16]. Figure 1 displays how the scientific research in the field of antimicrobial nanomaterials has grown exponentially over the last decade with the expectancy of achieving an effective solution against infection. Among the proposed mechanisms, the main approaches emphasize on two alternatives: (i) Preventing the adhesion of bacteria to avoid biofilm formation, or (ii) destroying the formed biofilm and eliminating bacteria without generation of AMR [17,18,19,20,21,22,23,24,25,26,27,28,29,30].

**Figure 2:**
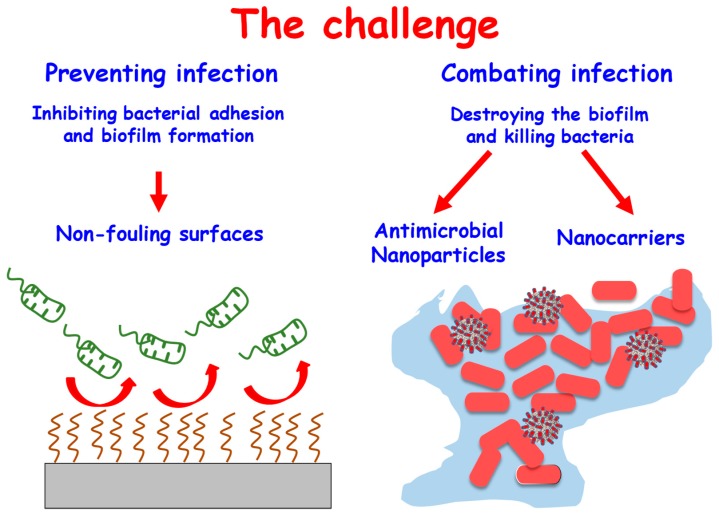
Two common infection-fighting strategies based on the design of nanostructured materials. The strategy to inhibit bacterial adhesion via surface modification is shown on the left. The use of nanosystems to destroy the formed biofilm using nanoparticles with intrinsic antimicrobial properties and nanoparticles acting as nanocarriers of different of antimicrobial agents are shown on the right.

An example of the first approach is the design of antibiofouling surfaces, made by altering their chemical and/or physical properties to make them highly unfavorable for the bacteria attachment and subsequent biofilm formation [31,32,33,34,35]. In this sense, the main requirement of this kind of surface in biomedical applications, particularly in bone tissue regeneration, is that at the same time the surface inhibits bacterial adhesion it also allows cell adhesion, which leads to integration of the bone implant or regeneration of the bone tissue [33,36]. Regarding the second approach, nanoparticles are successfully used to combat infection [10,20,21,25]. These nanomaterials have been used from two flanks, i.e., those nanoparticles that intrinsically possess antimicrobial effect [37] or those that are nanocarriers of antibiotics so a localized high concentration of the drug can be released at the site of infection [38]. The purpose of this review is to summarize recently published work on nanomaterials and their therapeutic potential for combating bacterial infection. Herein, we focus on inorganic-based nanomaterials, which show advantages compared to their organic counterparts, including their high thermal, chemical, and mechanical stabilities under physiological conditions and good biocompatibility. We provide a new viewpoint by dividing antibacterial nanomaterials into two categories: Materials to prevent the bacteria adhesion by the design of nonfouling surfaces and materials to extinguish the infection because they bear antibacterial properties or nanoparticles serving as vehicles for antimicrobial moieties. The activity of various inorganic-based nanomaterials against planktonic bacteria and biofilms will be discussed as well as their mechanism of action and potential toxicity. Figure 2 represents the challenge of this review manuscript.

## 2. Preventing the Bacterial Adhesion

Implant-related infection encompasses a complex biological process, which encloses an initial step of bacteria adhesion and subsequent biofilm formation. Bacterial adhesion is divided into two stages. The first one is reversible, and it is characterized by nonspecific interactions between the bacteria wall and implant surface, while the second step involves specific and nonspecific interactions mediated by proteins, which guide the adhesion to an irreversible state. In fact, once the biofilm is formed, bacterial eradication becomes insurmountable, being a difficult task to treat due to biofilm impermeability to antimicrobial agents and the immune system. To date, no treatment can guarantee the rapid and complete destruction of the biofilm, which constitutes a global challenge due to the fact that medical devices cannot yet actively resist bacterial adhesion, colonization, and biofilm formation. By consensus, inhibition of bacterial adhesion on the surface of an implant is one of the key strategies to prevent infection [39]. Different research groups work on the design of surfaces that inhibit the biofilm formation, which can be achieved by repelling bacterial adhesion or by killing approaching bacteria via direct impregnation with antibiotics, immobilization of bactericidal agents, or coating with antimicrobial moieties such as copper, silver, NO-releasing materials, and titanium oxide films.

As it has been mentioned, for bone implant-devices, the osseointegration is decisive to achieve their long-term survival. Thus, the efforts are addressed at the design of surfaces that inhibit the adhesion of bacteria at the same time as allowing the adhesion of eukaryotic cells for an adequate osseointegration [40]. Within this surface design, chemical and structural modification are relatively simple methodologies that can be performed without altering the properties of the implant itself [32]. Between the different strategies, our research group has focused on both chemical modifications providing a zwitterionic nature to the biomaterial surfaces and textural modification by tailoring the nanostructure surface.

### 2.1. Chemical Modifications to Create Zwitterionic Surfaces 

The zwitterionization consists of a simple method based on covalent grafting of different moieties resulting in surfaces with equal numbers of positive and negative charges, therefore maintaining overall electrical neutrality. It results in highly hydrophobic surfaces because a closely bound water layer forms a physical and energetic obstacle that inhibits the bacterial adhesion. These surfaces, the preparation of which has emerged as a groundbreaking strategy, are characterized by a high resistance to nonspecific protein adsorption, bacterial adhesion, and biofilm formation. Although there is great controversy in specifying the term zwitterion, since by IUPAC definition “zwitterionic surfaces are a subclass of polyampholites that have an equal number of non-ionizable positive and negative charges in the same group of pendants”, there are numerous strategies to confer zwitterionic-like behavior to material surfaces [41]. In general, the efforts have been focused on the covalent grafting to the surfaces of zwitterionic polymers or poly(sulfobetaine) and poly(carboxybetaine) derivatives containing mixed positively and negatively charged moieties within the same chain [42,43,44,45]. Another strategy would be the direct functionalization with low-molecular weight moieties bearing the same number of negative and positive charges. In this case, some amino acids can be used due their biocompatibility but they cannot be rigorously considered as zwitterions due to the presence of ionizable groups, providing a nonpermanent or pH dependent zwitterion-like action at the isoelectric point [46,47]. However, this could be taken as an advantage since the infectious process (the environment) is enclosed in certain conditions of acidity compared to normal physiological conditions. Thus, the lysine amino acid has been successfully used as a functional moiety to create zwitterionic surfaces on mesoporous bioactive glasses. The functionalized materials successfully presented bacterial antiadhesive properties for *Staphylococcus aureus* (*S. aureus*) and in vitro cytocompatibility behavior in MC3T3-E1 preosteoblast cells [48]. Another important strategy which has been developed is the design of zwitterionic-like surfaces on nanobiomaterials by simultaneous direct grafting of two organosilanes positively and negatively charged, respectively. In this case, it is possible to tailor the zwitterionic-like features by adjusting the molar ratio of the different reactants. In this sense, zwitterionic silica-based mesoporous bioceramics of SBA-15 type containing -NH_3_^+^/COO^−^ groups have been reported [49,50]. The zwitterionic nature was conferred by the cocondensation method using 3-aminopropyltriethoxysilane and carboxyethyl silanetriol sodium salt silanes, as -NH_3_^+^ and COO^−^ sources respectively, during the SBA-15 synthesis. The zwitterionic nature was achieved at pH values around 5.5 as determined through ζ potential studies of the isoelectric point. In this case, its behavior against bacteria was determined in severe inflammation conditions, i.e., pH equal to 5.5. The capability to inhibit the bacterial adhesion was tested by using *Escherichia coli* (*E. coli*) as Gram-negative bacteria showing a reduced bacterial adhesion (around 93%) with respect to bare pure silica SBA-15 material. Furthermore, human Saos-2 osteoblasts culture was used in order to determine the biocompatibility at physiological pH, showing an adequate behavior in these eukaryotic cells. Moreover, by taking advantage of the mesoporous structure, a zwitterionic SBA-15 type bioceramic with dual antibacterial ability has been prepared. In this case, in addition to having created the zwitterionic surface, the pores have been loaded with a broad-spectrum antibiotic to completely eradicate the biofilm. In this particular case, zwitterionic SBA-15 material was designed by the cocondensation route using alkoxysilane containing primary and secondary amine groups, N-(2-am-inoethyl)-3-aminopropyl-trimethoxysilane. Thus, the zwitterionic features are created from -NH_3_^+/^-SiO- and = NH_2_^+^/-SiO^−^ zwitterionic pairs present on the material surface, showing a 99.9% of inhibition of *S. aureus* after 90 minutes of incubation. At the same time, the presence of cephalexin inside of the mesopores leads to a sustained and controlled release for 15 days of incubation, which would help to eradicate the planktonic bacteria from the surroundings [51].

Additionally, metals widely used in clinics, such as Ti6Al4V alloy, also have been subjected to the zwitterionization process [52]. Previously, the metal surface was coated with nanocrystalline apatite layer to stimulate its bioactivity and to create an easier functionalizable surface. In this case, the zwitterionic nature is conferred by the postgrafting route using the silanes 3-aminopropyltriethoxysilane and carboxyethyl silanetriol sodium salt as –NH_3_^+^ and –COO^−^ sources respectively, in anhydrous conditions [53]. The in vitro bacteria test against *S. aureus* displayed a notable inhibition of bacterial adhesion and no biofilm formation. At the same time, these metal zwitterionic surfaces allowed a good osteoblast colonization and proliferation in preosteoblast MC3T3-E1 culture.

Finally, to date, these zwitterionic surfaces on biomaterials have not been clinically tested and just in vitro tests have been reported. A more in-depth study including in vivo assays should be reported to move these surfaces to the clinic.

### 2.2. Textural Modifications to Tailor the Nanostructure Surface

It is well known that both nanotopography and nanostructure of the surface play a crucial role in bacterial adhesion and biofilm formation [54,55]. Consequently, different approaches have been investigated to achieve artificial antibacterial surfaces based on the fabrication of different nanopatterns as nanotubes, nanoparticles, and nanopillars. In this sense, a TiO_2_ nanotubes coating has been applied onto titanium surfaces through the anodization route. Their antibacterial degree is strongly associated with the nanotube size, crystallinity (rutile or anatase phase), and contact angle [56,57,58,59]. Another example is the use of magnetron sputtering (MS-GLAD) on the surface of titanium alloys which produces nanostructure coatings in a large area with a variety of morphologies [60]. Recently, a nanopattering coating on Ti6Al4V substrates has been reported. The coating is formed by almost vertically aligned nanocolumns with lengths of 250–350 nm and diameters of 40–60 nm, separated from center to center by 100–200 nm [61]. These dense and highly packed nanotopography confer a superhydrophobic behavior with a contact angle of 102° (from 56° for the uncoated Ti6Al4V substrate). The antibacterial properties against different strains of *S. aureus* showed a decrease of the 70% in bacterial adhesion after 90 min of incubation. As the most important result of this study, it was demonstrated for the first time that these nanosurfaces inhibit the formation of biofilm after 24 hours of incubation, i.e., no protective cover (characteristic of biofilm) was formed, and only certain isolated bacteria appeared on the surface of the biofilm. In this sense, the mucopolysaccharide coverage characteristic of bacterial biofilm was not detected when stained by calcofluor. Simultaneously, in vitro biocompatibility assays with HOS cell line culture were performed. Osteoblast-like cells showed similar behavior in both surfaces (nanopattering coated and bare Ti alloy) with well-spread osteoblasts and adequate cell colonization, good adhesion, and appropriated cell proliferation and differentiation.

## 3. Nanomaterials with Unique Features as Potential Weapons to Fight Infections

As it has been commented in the introduction section, currently, cancer is the main area of nanoparticle applications although research is also being carried out in other therapeutic areas such as osteoporosis, cardiovascular diseases, Alzheimer’s disease, and infection [10,12,16,62]. In particular, the use of nanoparticles for infection treatment is motivated because the conventional antimicrobials fail due to AMR and the impenetrable biofilm formation, as well as the absence of novel drugs under expansion [15]. Furthermore, many bacteria are located intracellularly in an active or latent state, making it difficult for antibiotics to access them.

Nanoparticles offer numerous advantages to overcome these problems. In this sense nanoparticles act against bacteria through mechanisms which differ from the standard mechanisms of action of antibiotics, making them extremely useful against bacterial infection avoiding the dreaded AMR. The antibacterial mechanisms of nanoparticles are related to oxidative stress, metal ion release, and non-oxidative mechanisms, and generally trigger the formation of reactive oxygen species (ROS), enzymatic inhibition, protein deactivation, DNA damage, or changes in gene expression as well as bacteria wall disruption [63,64,65]. Furthermore, the multiple modes of action of nanoparticles significantly reduce the possibility of bacteria gaining resistances [66,67,68,69].

The production of ROS or oxygen free radicals, such as hydrogen peroxide (H_2_O_2_) or superoxide anions O_2_^−^, is indirectly induced by metal nanoparticles themselves. The excessive production of ROS by nanoparticles leads to a disturbed redox homeostasis and severe oxidative stress damaging cellular components, affecting membrane lipids, and altering the structure of DNA and proteins [70,71,72,73]. Moreover, metal-based nanoparticles gradually release metal ions that reach the intracellular compartment and interact with amino (-NH), mercapto (-SH), and carboxyl (-COOH) functional groups of proteins and nucleic acids [64,74,75]. As a result, several toxic effects can be produced such as protein coagulation, alteration in proteins related to electron transfer chains or deregulation of bacterial metabolic processes though impeded enzymatic activity. ROS production is also catalyzed by metallic ions leading to bacterial lipids and DNA damage. Interaction of the nanoparticles with the bacteria wall and membrane lead to nonoxidative mechanisms [76]. Bacterial resistance is largely based upon the structure of their cell wall and membrane, which are defensive barriers against environmental aggressions. Thus, damage of the cell wall leads to disrupted intracellular homeostasis and compromised bacterial function which causes mortality [64].

Gram-positive bacteria possess a rigid cell wall composed of a thin layer of peptidoglycans comprising carbohydrate polymers cross-linked through peptide residues [77]. Conversely, Gram-negative bacteria contain a thinner, more rigid peptidoglycan layer with much shorter cross-links, surrounded by a lipid membrane with lipopolysaccharides (LPS) forming a barrier presented on the surface [78]. The bacteria membrane components provide different adsorption pathways for the nanoparticles [79]. The negative charge on the surface of the bacteria wall can provide electrostatic interaction with positively charged nanoparticles that can accumulate, disturbing metabolic processes or causing perforation and even membrane leakage [24]. Silver or gold nanoparticles specifically interact with sulphur-containing constituents within the cell membrane, impeding cell wall synthesis [80,81,82].

### 3.1. Nanoparticles with Inherent Antibacterial Properties

The intrinsic antibacterial properties of some metals, metallic oxides, and metallic salts have been known for centuries, therefore being used to treat bacterial and fungal infections prior to the discovery of penicillin by Sir Alexander Fleming [20,83]. Although their medicinal utility was diminished with the antibiotic era, the actual emergence of AMR has led to the recovery of these earliest antimicrobial agents. Among metal and metal oxide nanoparticles, silver nanoparticles are probably the most promising of all the inorganic nanoparticles as a treatment for bacterial infections. Nevertheless, besides Ag, other metal nanoparticles such as Au, and metal oxide nanoparticles such as zinc oxide, copper oxide, iron oxide, and titanium dioxide, among others, are being intensively studied for antimicrobial treatment [83,84]. Some recent examples of this metal-based nanoparticles NPs are described in this section.

#### 3.1.1. Silver Nanoparticles

Although silver nanoparticles (AgNPs) are the most intensely considered metal nanomaterial for antimicrobial treatment [85], the understanding of their precise mechanism of action upon microbes remains incomplete. Multiple mechanisms may be involved [86], such as direct interaction of AgNPs with the bacterial membrane inhibiting cell wall synthesis or causing pits leading to cell lysis [87,88,89]. Moreover, silver oxidation in the biological media releases Ag^+^ ions continuously [90], which are bonded to thiol-containing proteins impairing their functions and also producing enhanced ROS generation [91,92,93,94]. While many attempts have been made to clarify the mode of action, the reported studies continue demonstrating their bactericidal efficacy. Hence, due to the potentiality of the AgNPs as bactericidal agents in clinical applications, another key point where many research efforts are being devoted is the synthetic methodology to prepare silver nanoparticles. Besides the traditional techniques based on the chemical reduction process, where a reducing agent for the Ag^+^ ion is used in the presence of stabilizers in a suitable solvent, new alternative approaches based on green chemistry are booming. The eco-friendly techniques incorporate the use of plants, biological, or microbial agents as reducing and capping agents. Silver nanoparticles obtained by green biogenic synthesis offer a novel potential alternative to chemically prepared nanoparticles [95].

Currently, AgNPs can be seen as an alternative treatment for some clinical situations due to their antimicrobial activity and wound healing effects. For example, AgNPs have been in vivo evaluated as a postsurgical treatment for *Caseous lymphadenitis* in small ruminants. The etiological agent of this disease is *Corynebacterium pseudotuberculosis*, a Gram-positive and facultative intracellular bacterium. In the experiment twenty-nine goats and sheep with clinical signs of *Caseous lymphadenitis* were surgically operated on to excise the caseous lesions that were treated with an ointment formulation based on AgNPs mixed with natural waxes and oils in the experimental group, or with the conventional treatment with 10% iodine in the control group. It could be concluded that postsurgical treatment of *Caseous lymphadenitis* using the AgNPs-based ointment led to faster healing, decreased wound contamination, and presented no apparent toxic effects [96]. Another field of clinical development of the AgNPs is related to orthopaedic implants. As above commented, for the treatment or prevention of implant-related infections, materials that exhibit antibacterial properties at the same time as promoting osteogenesis are required. AgNP coatings of implants must take into account the dose-dependent cytotoxicity of silver and its negative impact on bone implants. In view of this remark, a bioinspired hybrid coating containing polydopamine, hydroxyapatite, AgNPs, and chitosan has been prepared on the surface of titanium implants. The double chelating effect of polydopamine and chitosan significantly reduces silver ion release from the AgNPs in the hybrid coating. The coating exhibits excellent antibiofilm efficiency of 91.7%, 89.5%, and 92.0% for *S. aureus*, *S. epidermidis*, and *E. coli*, respectively. In addition, the coating can significantly stimulate osteogenic differentiation of MC3T3-E1 cells and promotes bone-implant osseointegration in vivo. Therefore, the hybrid coating exhibits antibacterial properties as well as allowing bone-implant osseointegration, thereby providing insights into the design of multifunctional implants for long-term orthopedic applications [97].

#### 3.1.2. Gold Nanoparticles

Metallic gold is stable against oxidation in biological mediums, which makes it nontoxic and a biocompatible metal. However, gold nanoparticles (AuNPs) indeed exhibit antimicrobial effects via different mechanisms [64,69]. AuNPs and Au nanoclusters possess catalytic activity analogous to various enzymes such as peroxidase, glucose-oxidase, and/or superoxide dismutase [98]. This enzyme-like activity led to an increased generation of ROS affecting bacteria through oxidative stress mechanism [99,100]. In addition, AuNPs can be irreversibly bound onto the thiol groups present on different proteins, for example in nicotinamide adenine dinucleotide (NADH) dehydrogenase, in this case affecting the reduction–oxidation balance within the bacterial respiratory chain and thus generating oxidative stress [101]. Perhaps one of the most recent and interesting applications of gold nanomaterials as bactericidal agents takes place when the physical properties of gold are exploited at the nanoscale. In this sense, AuNPs possess excellent photothermal properties since their plasmon resonance makes them able to absorb light in the near infrared (NIR) window, and in turn generates heat that can be used for ablation of bacteria or disruption of biofilms [102]. Recently, a nonantibiotics-based nanoformulation containing Au nanorods has shown a remarkable antibacterial efficacy in treating drug-resistant pneumonia when applied in combination with NIR photothermal treatment. The 50-100 nm long gold nanorods are decorated with glycomimetic polymers to specifically block bacterial lectins which are essential for bacterial biofilm development. This novel formulation shows the most efficient bacteria inhabitation and killing against *Pseudomonas aeruginosa* infection, through lectin blocking and the NIR light-induced photothermal effect of gold nanorods [103].

#### 3.1.3. Metal Oxide Nanoparticles

Metal oxide nanomaterials such as zinc oxide (ZnO), iron oxide (Fe_3_O_4_), copper oxide (CuO), magnesium oxide (MgO), and titanium dioxide (TiO_2_) nanoparticles are known to possess antimicrobial activity over a range of Gram-positive and Gram-negative bacteria, including resistant bacterial strains [84,104]. Their antibacterial activity is usually related to the generation of ROS, attributed to their intrinsic photocatalytic activity or to the release of the metallic ions [105,106].

**Figure 3:**
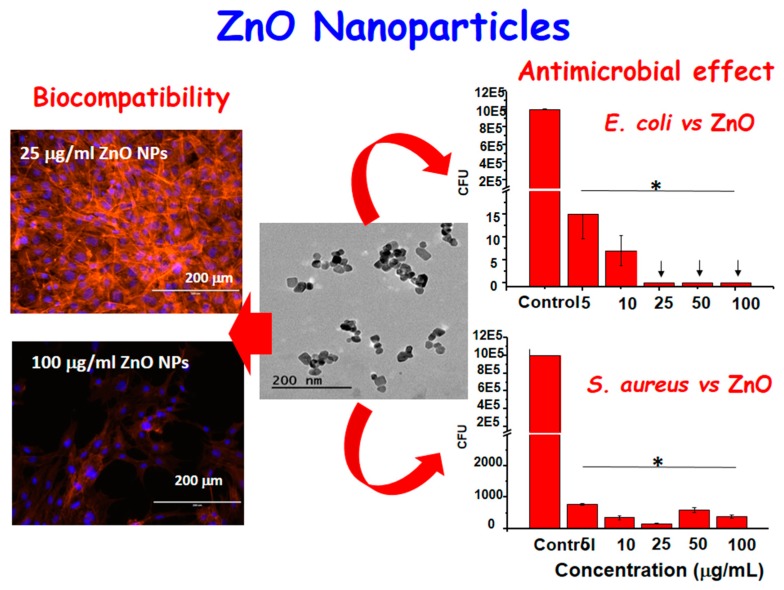
Biocompatibility and antimicrobial effect of ZnO nanoparticles prepared by microwave-assisted synthesis. A TEM image corresponding to the ZnO nanoparticles, round-shaped, of 20 nm in diameter is shown in the center. (**Left**) Confocal images corresponding to preosteoblast cultured up to 70% of confluence after incubation for 4 days with ZnO nanoparticles at different concentrations, showing a good biocompatibility. (**Right**) Antimicrobial effect against *E. coli* and *S. aureus* in planktonic stage incubated for 24 h in the presence of different concentrations of ZnO nanoparticles. The reduction of colony forming units (CFU) is represented (*p* < 0.05, significant differences compared to control denoted by an asterisk (*)). The arrows denote an absolute 100% of efficacy.

Recently, amine functionalized ZnO nanocrystals have been designed as a highly biocompatible and osteoinductive nanoantibiotic agent for bone tissue engineering. The ZnO nanocrystals of 20 nm in diameter have been prepared with a novel, fast, and reproducible microwave-assisted synthesis. After chemical functionalization by anchoring aminopropyl groups onto the ZnO surface, the ZnO-NH_2_ nanocrystals were tested in terms of biocompatibility, promotion of cell proliferation, and differentiation towards preosteoblast cells, and also in terms of antimicrobial activity against Gram-positive and Gram-negative bacteria, such as *E. coli* and *S. aureus*, respectively (see Figure 3). The in vitro results suggest that ZnO-NH_2_ nanocrystals are a promising candidate to solve infectious diseases in bone implants and at the same time promote bone tissue proliferation [107].

Antimicrobial activity of iron oxide nanoparticles (IONPs)-based nanosystems against different microorganisms has been already recently reviewed. One of the main mechanisms of action by which systems based on IONPs generate bacteria toxicity is ROS generation through the Fenton reaction [108]. However, by taking advantage of the magnetic properties of IONPs, alternative physical antibacterial strategies can be proposed to fight against AMRs. For example, multiple drug resistant *S. aureus* and uropathogenic *E. coli* have been trapped into positively charged magnetic core-shell nanoparticles by electrostatic interaction. All the trapped bacteria could be completely killed within 30 minutes when exposed to a radiofrequency current owing to the loss of membrane potential and dysfunction of membrane-associated complexes. This physical treatment kills pathogenic bacteria and blocks biofilm formation without leading to antibiotic resistance [109]. Another research work used IONPs loaded with nisin, a known bacteriocin, which is commonly inefficient against Gram-negative bacteria. The IONPs were activated by high pulsed electric and electromagnetic fields to induce additional permeabilization and local magnetic hyperthermia. The results on the assays on the Gram-positive *Bacillus subtilis* and Gram-negative *E. coli* showed that the high pulsed magnetic fields increase the antimicrobial efficiency of nisin loaded on the IONPs, similar to electroporation or magnetic hyperthermia methods, resulting in a synergistic treatment [110].

### 3.2. Nanomaterials as Nanocarriers: Mesoporous Silica Nanoparticles

An alternative strategy to fight infection with nanoparticles is to use them as vehicles to deliver antimicrobial agents such as antibiotics or other bactericide nanoparticles. Nanocarriers of antimicrobial agents should be able to shield the active compound from degradation and to enhance the potency of the active compound or improve its bioavailability for treatment. The nanocarrier may enable controlled and sustained release of the loaded antimicrobial drugs, which is useful for maintaining an optimum level of drug concentration in the bloodstream for a period of time. They also may offer the possibility to simultaneously deliver several antibiotics or to act in a combined therapy if using other stimuli responsive nanoparticles in the same nanosystem. Moreover, the nanocarriers may also provide a platform for surface modification that allows specific targeting to the site of infection, only once an infection has occurred.

**Figure 4:**
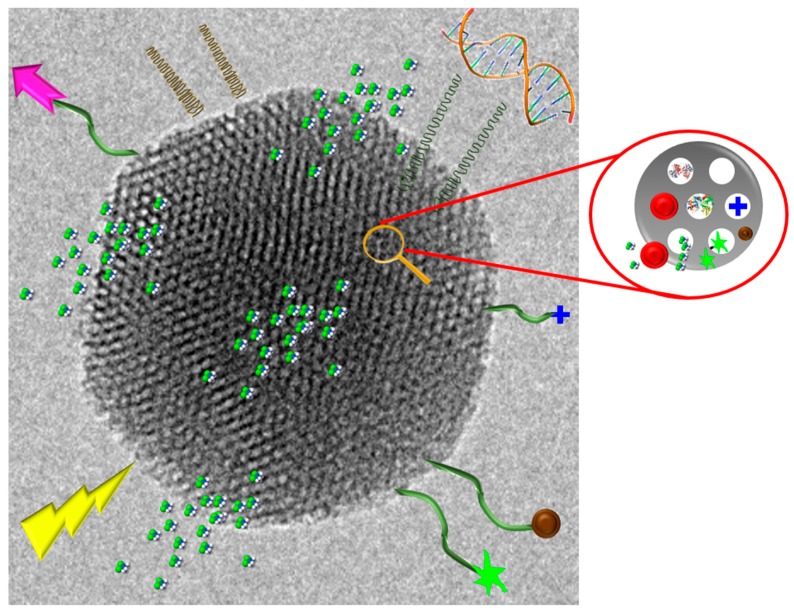
Schematic representation of the versatility and functionality of mesoporous silica nanoparticles (MSNs. TEM) image corresponding to an MSN of 150 nm in diameter showing the mesoporous arrangement in the 2D hexagonal structure (*p6mm* plain group). On the image, cartoons represent the drug loading capability, active targeting, and stimuli-responsive possibilities of MSNs.

Among the different, materials which can compose these nanocarriers, mesoporous silica nanoparticles (MSNs) constitute one of the most promising due to their interesting properties of advanced inorganic nanoplatforms as drug delivery systems. The main strengths of MSNs are high loading capacity, biocompatibility, ease of production, and high degree of tunability regarding size, morphology, and pore diameter. Furthermore, MSNs can be easily synthesized on a large scale, showing a great variety of morphologies and surface functionalities using different strategies [10]. Figure 4 shows a schematic representation of the versatility and functionality of MSNs regarding their biomedical applications. Initially, these nanocarriers have shown high interest in cancer treatment due to the wide versatility in their functionalization, being able to design smart nanomaterials with stimulus responsive components [10,111], possessing also cancer cell targeting capability [112] and penetrability towards the deepest areas of solid tumors [113]. Recently, this technology has been also successfully applied to osteoporosis treatment in an animal model [12].

In this sense, effective new alternatives for the management of bone infection can be achieved through the development of antibiotic nanocarriers able to penetrate bacterial biofilm, thus enhancing antimicrobial effectiveness. An example of this kind of nanosystem, also denoted as “nanoantibiotic”, exists in MSNs loaded with levofloxacin (LEVO) as antimicrobial agent externally functionalized with N-(2-aminoethyl)-3-aminopropyltrimethoxysilane as a targeting agent. This amine functionalization provides MSNs of positive charges, which improves the affinity towards the negatively charged bacteria wall and biofilm. After physicochemical characterization, “in vial” LEVO release profiles and the in vitro antimicrobial effectiveness of the different released doses were investigated. The efficacy of this nanoantibiotic against a *S. aureus* biofilm was also determined, showing practically total destruction of the biofilm due to the high penetration ability of the developed nanosystem. These findings open up promising expectations in the field of bone infection treatment [114].

**Figure 5:**
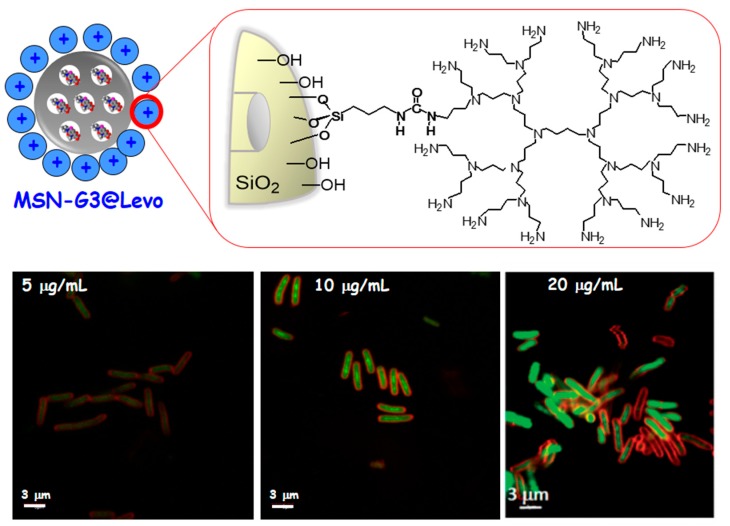
MSN as nanocarrier of antimicrobial agent (levofloxacin) and functionalized with a bacteria membrane targeting agent (poly(propyleneimine) dendrimer of third generation, G3). In this case the functionalization of MSNs with G3 macromolecules increase the internalization in bacteria Gram negative (*E. coli*), which is dosage dependent as it can be observed in the confocal images. The synergistic combination of polycationic dendrimers as bacterial membrane permeabilization agents with LEVO-loaded MSNs triggers an efficient antimicrobial effect on *E. coli* biofilm. These results open up very promising prospects for their potential application as new anti-infective therapies. In the confocal images the red color represents the bacteria membrane and the green color represents the labelled-MSN materials.

Another important strategy is to provide an effective and novel solution for the treatment of infection by using nanovehicles loaded with antibiotics capable of penetrating the bacterial wall, thus increasing the antimicrobial effectiveness. In this case these “nanoantibiotics” were composed of MSNs, which acted as nanocarriers of LEVO localized inside the mesopores. To provide the nanosystem of bacterial membrane interaction capability, a polycationic poly(propyleneimine) dendrimer of third generation (G3) was covalently grafted to the external surface of the LEVO-loaded MSNs. After physicochemical characterization of this nanoantibiotic, the release kinetics of LEVO and the antimicrobial efficacy of each released dosage were evaluated. Besides, internalization studies of the MSNs functionalized with the G3 dendrimer were carried out, showing a high penetrability throughout Gram-negative bacterial membranes (see Figure 5). This work evidences that the synergistic combination of polycationic dendrimers as bacterial membrane permeabilization agents with LEVO-loaded MSNs triggers an efficient antimicrobial effect on Gram-negative bacterial biofilm. These positive results open up very promising expectations for their potential application in new infection therapies [115]. 

**Figure 6:**
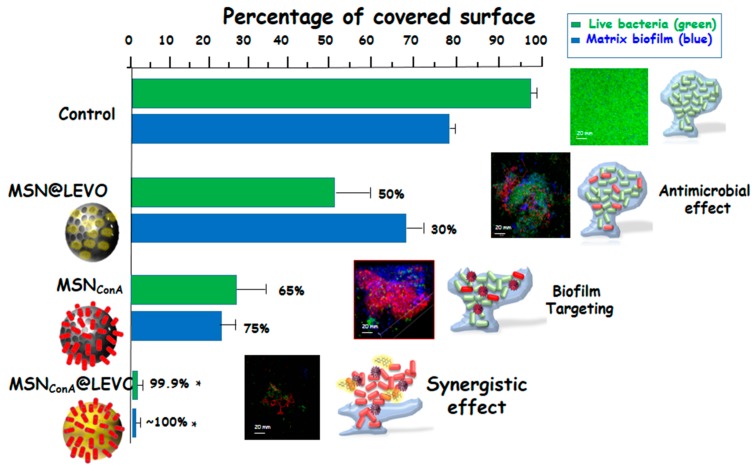
MSN as nanocarrier of antimicrobial agent (levofloxacin) and functionalized with a biofilm targeting agent (Concanavaline A, ConA). In this case the functionalization of MSNs with ConA favours its internalization in *E. coli* biofilms affording a synergistic combination with LEVO-loaded MSNs, which triggers an efficient antimicrobial effect on *E. coli* biofilm. The image represents the percentage of covered surface by live bacteria (green) and mucopolysaccaride layer (blue) and the representative confocal images show a complete reduction after incubation with the nanosystems functionalized with ConA and loaded with levofloxacin (MSNConA@LEVO).

Finally, the ability of bacteria to form biofilms hinders any conventional treatment for chronic infections and has serious socio-economic implications. For this purpose, a nanocarrier capable of overcoming the barrier of the mucopolysaccharide matrix of the biofilm and releasing its loaded-antibiotic within this matrix would be highly desirable. Herein, we have developed a new nanosystem based on LEVO-loaded MSNs decorated with the lectin concanavalin A (ConA). The presence of ConA promotes the internalization of this nanosystem into the biofilm matrix, which increases the antimicrobial efficacy of the antibiotic hosted within the mesopores (see Figure 6). This nanodevice is envisioned as a promising alternative to conventional treatments for infection by improving the antimicrobial efficacy and reducing side effects [116].

## 4. Conclusions and Futures Perspectives

The increased antibiotic resistance, and consequently the formation of biofilms, have resulted in a critical problem for the health industry, due to the ineffectiveness of the conventional antimicrobial therapies. Nanomaterials display a promising technology to solve these issues. This review tried to give an overview of the different solutions according to two different approaches: To prevent the infection via the modification of nanomaterial surfaces or to combat the infection by the accurate design of nanoparticles with inherent antimicrobial features or nanoparticles as carriers of antimicrobial agents. Both surface zwitterionization and nanostructured coatings were presented as highly powerful tools for the prevention of bacterial adhesion and biofilm formation. These surfaces show a good degree of biocompatibility, which is very important when bringing this type of technology to the clinic. Metal and metal oxide nanoparticles show effective antimicrobial activity with rapid time-kill and evading of antibiotic resistance based on their specific properties at the nanoscale and their multiple mechanisms of action. Mesoporous silica nanoparticles used as nanocarriers offer extraordinary advantages by being able to functionalize their surface with targeting agents and considerably increase the activity of the loaded antimicrobial agent. This is certainly the starting point towards a considerable improvement in conventional treatments, where the tendency is to combine all the elements in order to effectively abolish the dreaded infections. At this point, for clinic translation, it is important to know about their safety and cytotoxicity, which has been addressed, in most of the cases, only in vitro in different cell cultures. However, in vivo models should be carried out to better understand the biological effect of the proposed nanosystems, comprising toxicity, metabolism, biodistribution, clearance, and mechanism of action for a good practice towards the clinical application.

## Abbreviations

AMR	Antimicrobial resistance
ConA	concanavalin A
AuNPs	gold nanoparticles
FDA	Food and Drugs Administration
IUPAC	International Union of Pure and Applied Chemistry
IONPs	iron oxide nanoparticles
LEVO	levofloxacin
LPS	lipopolysaccharides
MS-GLAD	magnetron sputtering
MSNs	mesoporous silica nanoparticles
ROS	reactive oxygen species
AgNPs	Silver nanoparticles
